# Calcium Binding Protein Ncs1 Is Calcineurin Regulated in Cryptococcus neoformans and Essential for Cell Division and Virulence

**DOI:** 10.1128/mSphere.00761-20

**Published:** 2020-09-09

**Authors:** Eamim Daidrê Squizani, Júlia Catarina Vieira Reuwsaat, Sophie Lev, Heryk Motta, Julia Sperotto, Keren Kaufman-Francis, Desmarini Desmarini, Marilene Henning Vainstein, Charley Christian Staats, Julianne T. Djordjevic, Lívia Kmetzsch

**Affiliations:** a Centro de Biotecnologia, Universidade Federal do Rio Grande do Sul, UFRGS, Porto Alegre, Rio Grande do Sul, Brazil; b Centre for Infectious Diseases and Microbiology, The Westmead Institute for Medical Research, Sydney, New South Wales, Australia; c Sydney Medical School-Westmead, University of Sydney, Sydney, New South Wales, Australia; d Marie Bashir Institute for Infectious Diseases and Biosecurity, University of Sydney, Sydney, New South Wales, Australia; University of Georgia

**Keywords:** calcium binding protein, calcium sensor, *Cryptococcus neoformans*, calcium signaling

## Abstract

Intracellular calcium (Ca^2+^) is crucial for signal transduction in Cryptococcus neoformans, the major cause of fatal fungal meningitis. The calcineurin pathway is the only Ca^2+^-requiring signaling cascade implicated in cryptococcal stress adaptation and virulence, with Ca^2+^ binding mediated by the EF-hand domains of the Ca^2+^ sensor protein calmodulin. In this study, we identified the cryptococcal ortholog of neuronal calcium sensor 1 (Ncs1) as a member of the EF-hand superfamily. We demonstrated that Ncs1 has a role in Ca^2+^ homeostasis under stress and nonstress conditions, as the *ncs1Δ* mutant is sensitive to a high Ca^2+^ concentration and has an elevated basal Ca^2+^ level. Furthermore, *NCS1* expression is induced by Ca^2+^, with the Ncs1 protein adopting a punctate subcellular distribution. We also demonstrate that, in contrast to the case with Saccharomyces cerevisiae, *NCS1* expression in C. neoformans is regulated by the calcineurin pathway via the transcription factor Crz1, as *NCS1* expression is reduced by FK506 treatment and *CRZ1* deletion. Moreover, the *ncs1Δ* mutant shares a high temperature and high Ca^2+^ sensitivity phenotype with the calcineurin and calmodulin mutants (*cna1*Δ and *cam1Δ*), and the *NCS1* promoter contains two calcineurin/Crz1-dependent response elements (CDRE1). Ncs1 deficiency coincided with reduced growth, characterized by delayed bud emergence and aberrant cell division, and hypovirulence in a mouse infection model. In summary, our data show that Ncs1 has a significant role as a Ca^2+^ sensor in C. neoformans, working with calcineurin to regulate Ca^2+^ homeostasis and, consequently, promote fungal growth and virulence.

**IMPORTANCE** Cryptococcus neoformans is the major cause of fungal meningitis in HIV-infected patients. Several studies have highlighted the important contributions of Ca^2+^ signaling and homeostasis to the virulence of C. neoformans. Here, we identify the cryptococcal ortholog of neuronal calcium sensor 1 (Ncs1) and demonstrate its role in Ca^2+^ homeostasis, bud emergence, cell cycle progression, and virulence. We also show that Ncs1 function is regulated by the calcineurin/Crz1 signaling cascade. Our work provides evidence of a link between Ca^2+^ homeostasis and cell cycle progression in C. neoformans.

## Abstract (precis)

Cryptococcus neoformans is the major cause of fungal meningitis in HIV-infected patients. Several studies have highlighted the important contributions of Ca^2+^ signaling and homeostasis to the virulence of C. neoformans. Here, we identify the cryptococcal ortholog of neuronal calcium sensor 1 (Ncs1) and demonstrate its role in Ca^2+^ homeostasis, bud emergence, cell cycle progression, and virulence. We also show that Ncs1 function is regulated by the calcineurin/Crz1 signaling cascade. Our work provides evidence of a link between Ca^2+^ homeostasis and cell cycle progression in C. neoformans.

## INTRODUCTION

Cryptococcus neoformans is a basidiomycetous pathogenic yeast found mostly in soil and bird droppings (1–3). This pathogen is the etiological agent of cryptococcosis, which affects mainly immunocompromised individuals. This disease affects more than 220,000 HIV-infected patients per year, resulting in more than 180,000 deaths worldwide (3, 4). The lung infection is initiated following the inhalation of small desiccated cells or spores. The infection can then spread via the bloodstream to the central nervous system, causing meningoencephalitis, which is the primary cause of death (1, 5). To survive within the host environment, C. neoformans produces several virulence determinants, including a polysaccharide capsule, the pigment melanin, secreted enzymes (6–9), and extracellular vesicles (10). C. neoformans survival in the host is possible only due to its ability to grow at 37°C and is also aided by its capacity to survive within phagocytic mammalian cells (1, 11–15).

Fungal fitness and survival in the host environment are controlled by numerous signaling pathways, including those that are regulated by intracellular Ca^2+^, which is an essential second messenger in eukaryotic cells (16–19). An increase in cytosolic Ca^2+^ is monitored by Ca^2+^ sensor proteins that, upon binding to Ca^2+^, change their conformation and transduce signals onto downstream targets (20, 21). An important Ca^2+^ sensor in fungal cells is calmodulin, which is a component of the calcineurin signaling pathway. Ca^2+^-induced conformational change in calmodulin activates the serine-threonine phosphatase calcineurin. Calcineurin then mediates the regulation of several cellular responses by initiating changes in the phosphorylation status of its downstream targets (18, 22, 23). A major target of cryptococcal calcineurin is the transcription factor Crz1, which regulates the expression of genes involved in stress response and in the maintenance of cell wall integrity (24, 25). In C. neoformans, the calcineurin pathway is also essential for growth at 37°C, sexual reproduction, and virulence (26); in Saccharomyces cerevisiae, it is required for cell cycle progression (27).

Given that high levels of cellular Ca^2+^ can be toxic, Ca^2+^ homeostasis is strictly regulated by several proteins acting as transporters, channels, or pumps (28). In C. neoformans, these proteins include Cch1, a Ca^2+^ voltage-gated channel essential for virulence, and Mid1, a stretch-activated Ca^2+^-channel, both found in the plasma membrane (19, 29). Other cryptococcal calcium transporters that also promote virulence include the Ca^2+^ ATPase EcaI, found in sarcoplasmic/endoplasmic reticulum, and the H^+^/Ca^2+^ exchanger protein Vcx1 and the Ca^2+^ ATPase Pmc1, both localized on vacuolar membranes and responsible for Ca^2+^ storage (30–33). Pmc1 is also required for C. neoformans transmigration through the blood-brain barrier (BBB), proving that Pmc1-regulated Ca^2+^ homeostasis is crucial for disease progression (33).

Despite the importance of Ca^2+^ homeostasis-related proteins in fungal cell fitness and virulence, with the exception of calmodulin, little is known about the function of other calcium binding proteins (CBPs) that act as Ca^2+^ sensors in C. neoformans. One such protein is neuronal calcium sensor 1 (Ncs1). Ncs1 orthologs in other fungi have roles in cell growth and viability, tolerance to Ca^2+^ (34–41), membrane sterol distribution, and expression of Ca^2+^ transporter genes (41). In this study, we identified and characterized the Ncs1 ortholog in C. neoformans. Using gene deletion and *in silico* analysis, we investigated the role of Ncs1 in Ca^2+^ homeostasis, growth, stress tolerance, and virulence and whether Ncs1 function is linked to the calcineurin pathway.

## RESULTS

### Identification of the Ncs1 ortholog in C. neoformans.

CBPs are either predominantly intrinsic membrane proteins that transport Ca^2+^ through membranes or Ca^2+^-modulated proteins, mainly represented by Ca^2+^ sensors involved in signal transduction (21, 28). The latter include calmodulin and calcineurin, which harbor the calcium-binding (EF-hand) domain. Both proteins have been well studied in eukaryotic cells, including C. neoformans (25, 26, 42). However, calmodulin is the only intracellular calcium sensor characterized so far in C. neoformans. Considering the number and complexity of processes regulated by Ca^2+^, we sought to identify other CBPs in the EF-hand superfamily with Ca^2+^ sensor functions. In this context, we searched for the neuronal calcium sensor 1 (Ncs1) homolog in C. neoformans, given that this protein is important for Ca^2+^ regulated processes in a variety of eukaryotic cells (43).

**FIG 1:**
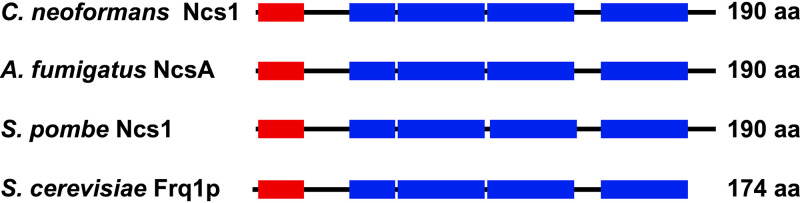
Identification of Ncs1 as a putative calcium binding protein in C. neoformans. Comparative *in silico* analysis of C. neoformans Ncs1 (CNAG_03370), Aspergillus fumigatus NcsA (Afu6g14240), Schizosaccharomyces pombe Ncs1 (SPAC18B11.04), and S. cerevisiae Frq1 (YDR373W) amino acid (aa) sequences indicates the presence and positions of the four EF-hand domains (blue bars) and the N-terminal myristoylation domain (red bars).

For this purpose, we performed an *in silico* analysis at FungiDB to identify the *NCS1* coding sequence in the C. neoformans H99 genome (CNAG_03370). Ncs1 is well conserved in eukaryotes, with orthologs sharing common regions, such as EF-hand domains and a myristoylation motif. Our analysis revealed that C. neoformans Ncs1 contains four predicted EF-hand domains that span the full length of the protein (Fig. 1). Moreover, the presence of an N-terminal myristoylation motif was identified using the NMT-themyr predictor database. Myristoylation, a lipid modification conserved among eukaryotic Ncs1 proteins (44), is important for cell signaling, protein-protein interaction, and protein targeting to endomembrane systems and the plasma membrane (45). Comparative analysis of the C. neoformans Ncs1 protein sequence with the products encoded by Aspergillus fumigatus *NCSA* (Afu6g14240), Schizosaccharomyces pombe *NCS1* (SPAC18B11.04), and S. cerevisiae *FRQ1* (YDR373W), which are already functionally characterized (34, 40, 41), revealed high degrees of amino acid sequence similarity (86, 87, and 81%, respectively) (Fig. 1).

### Disruption of the *NCS1* gene affects C. neoformans traits associated with calcium homeostasis.

**FIG 2:**
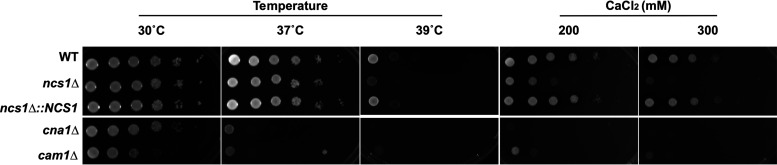
Disruption of *NCS1* leads to stress sensitivity in C. neoformans. Spot plate assays of the WT, *ncs1Δ* mutant, and *ncs1Δ*::*NCS1* complemented cells were performed on YPD agar. The plates were incubated at 30°C (control for normal growth), 37°C, or 39°C and under stress induced by Ca^2+^ (200 mM or 300 mM CaCl_2_ at 30°C). The calcineurin (*cna1*Δ) and calmodulin (*cam1Δ*) mutants were included as controls, separated by a thin white line that indicates noncontiguous portions of the same image. All assays were conducted for 48 h.

Calcium sensor proteins measure fluctuations in free cytosolic Ca^2+^ and transduce the signal to downstream effectors (41, 42, 46). To determine whether Ncs1 plays a similar role in C. neoformans, we obtained a *NCS1* gene knockout strain (*ncs1Δ*) from Madhani’s mutant collection (47) and generated an *NCS1* reconstituted (*ncs1Δ*::*NCS1*) strain (see Fig. S1 in the supplemental material) using the *ncs1Δ* background. We then evaluated the ability of these mutant strains to grow under different stresses. We initially chose high Ca^2+^ concentration (to alter Ca^2+^ homeostasis) and high temperatures (37°C and 39°C), as the calcineurin (*cna1Δ*) and calmodulin (*cam1Δ*) mutants were shown to be sensitive under these growth conditions (24, 25, 42). We observed impaired *ncs1Δ* strain growth in high Ca^2+^ levels and at 39°C but not at 37°C; these growth defects were restored to wild-type (WT) levels in the *ncs1Δ*::*NCS1* strain (Fig. 2). Lower Ca^2+^ concentrations (ranging from 1 to 20 mM CaCl_2_) did not influence *ncs1Δ* strain growth (data not shown). Other traits associated with the Ca^2+^-calcineurin pathway, such as growth in the presence of cell wall-perturbing agents (calcofluor white and Congo red) and osmotic stress (1 M NaCl) were evaluated in the *ncs1*Δ null mutant, with no effect observed (Fig. S2).

10.1128/mSphere.00761-20.3 FIG S1 Confirmation of mutant genotypes. (A) The corrected integration of inactivation cassette to generate the *ncs1Δ* null strain was evaluated employing PCR with a primer pair that amplifies a portion inside the coding region. As loading control, a fragment of the *ACT* gene was amplified. (Left) Confirmatory PCR analysis. NC, negative control. (Right) Diagram representing the double crossover at the WT *NCS1* locus. (B) Integration of the complementation cassette into the inactivated *ncs1Δ* locus was evaluated with PCR using primers that hybridize inside the complementation cassette (CDSR and G418F primers) and at chromosomal sites outside the double recombination location (5′UTRF and 3′UTRR primers). Each primer pair was used independently to evaluate correct integration at the *NCS1* CDS upstream site (5′UTRF and CDSR primers), as well as the *NCS1* CDS downstream site (G418F and 3′UTRR primers). (Left) Confirmatory PCR analysis. NC, negative control. (Right) Diagram representing the double crossover at the *ncs1Δ* locus. (C) Evaluation of the correct integration of the *NCS1*::*GFP* cassette into the WT *NCS1* locus was performed using two primer pairs (NCS1OF plus NCS1GFPovR and G4183UTRovF plus NCS1OR) independently to assess correct integration at the *NCS1* CDS upstream site and the *NCS1* CDS downstream site, respectively. (Left) Confirmatory PCR analysis. (Right) Diagram representing the double crossover at the WT *NCS1* locus. (D) Evaluation of the correct integration of the inactivation cassette of the *MID1* gene in the *ncs1Δ* strain was performed using PCR with a primer pair that amplifies a region inside the coding region. As a loading control, a fragment of the *ACT* gene was amplified. (Left) Confirmatory PCR analysis. (Right) Diagram representing the double crossover at the *MID1* locus. (E) Evaluation of transcript levels of *NCS1* (left gels) or *MID1* (right gels) in distinct mutants and in the WT strain was conducted using RT-PCR with RNA isolated from yeast strains grown in YPD for 24 h. As loading control, the transcript levels of the *ACT1* gene were also evaluated. Download FIG S1, TIF file, 1.7 MB. Copyright © 2020 Squizani et al. 2020 Squizani et al. This content is distributed under the terms of the Creative Commons Attribution 4.0 International license.

10.1128/mSphere.00761-20.4 FIG S2 Phenotypic analysis of the *ncs1Δ* null mutant strain. The indicated strains were evaluated by spot plate assay under different stress conditions: altered temperature, saline stress (NaCl at 1 M), low phosphate and oxidative (menadione) stresses, and cell wall (calcofluor white and Congo red) stress. Pictures were taken after 48 h of incubation. The *cna1Δ* calcineurin mutant was included to assess the degree of phenotypic overlap with *ncs1Δ*. Download FIG S2, TIF file, 1.0 MB. Copyright © 2020 Squizani et al. 2020 Squizani et al. This content is distributed under the terms of the Creative Commons Attribution 4.0 International license.

**FIG 3:**
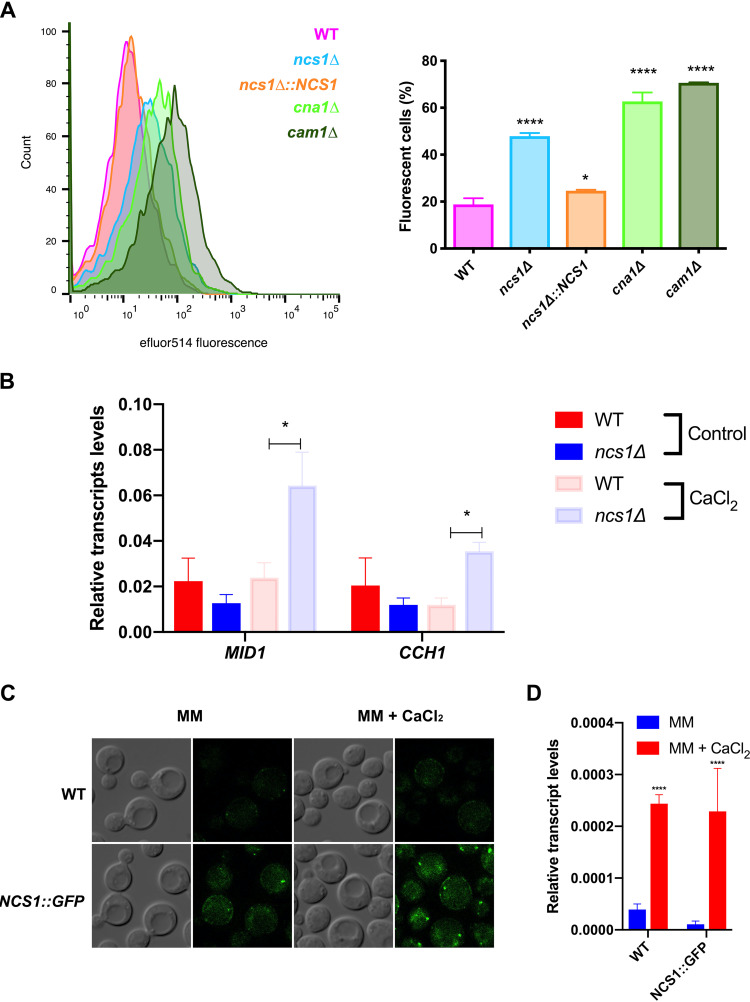
Cryptococcal Ncs1 is associated with Ca^2+^ homeostasis. (A) The basal levels of free intracellular Ca^2+^ in WT, *ncs1Δ*, *ncs1Δ*::*NCS1*, *cna1Δ*, and *cam1Δ* mutant cells were quantified by flow cytometry following staining with the calcium-specific dye Fluo-4-AM. The left side represents the histogram of Fluo-4-AM emitted fluorescence of indicated strains cultivated in YPD medium at 30° C. The right side represents the percentage of gated fluorescent cells ± standard deviation (three biological replicates). Mean values were compared using one-way ANOVA and Dunnett’s *post hoc* test. Statistical significance is represented as follows: ****, *P* < 0.0001, and *, *P* < 0.05. (B) The transcript levels of genes encoding the calcium transporters, *CCH1* and *MID1*, were evaluated using RT-qPCR. The WT and *ncs1Δ* strains (10^6^ cells/ml) were incubated in YPD for 16 h with shaking, either at 37°C (control) or at 37°C supplemented with Ca^2+^ (100 mM CaCl_2_). RNA was extracted and cDNA synthesized. Each bar represents the mean ± the standard deviation (*n* = 3) for each gene in each strain normalized to actin. Statistical analysis was performed using Student’s *t* test (*, *P* < 0.05). (C) Ncs1 was tagged with GFP (*NCS1*::*GFP*), and the effect of CaCl_2_ supplementation on Ncs1 production and subcellular localization was assessed by fluorescence microscopy. YPD overnight cultures of the WT (autofluorescence background control) and the *NCS1*::*GFP* strain were washed twice with water and used to seed on minimal medium (MM) or MM supplemented with Ca^2+^ (100 mM CaCl_2_) at an OD_600_ of 1. The cultures were further incubated for 4 h at 30°C prior to visualization. DIC and green fluorescent images are included. (D) The cultures prepared for panel C were also used to extract RNA and perform RT-qPCR to assess the effect of Ca^2+^ on the transcript levels of *NCS1*, normalized to actin. Statistical analysis was performed using one-way ANOVA with Tukey *post hoc* test. Comparisons were conducted between WT cells grown in the absence or in the presence of Ca^2+^ or between *NCS1*::*GFP* cells grown in the absence or in the presence of Ca^2+^. ****, *P* < 0.0001.

We also evaluated whether the level of free intracellular Ca^2+^ in C. neoformans is affected in the absence of Ncs1. Relative to the WT strain, the *ncs1Δ* mutant had a higher basal level of free cytosolic Ca^2+^, which was reduced to WT levels in the *ncs1Δ*::*NCS1* strain. This high-Ca^2+^-level phenotype was shared with that observed for the *cna1Δ* and *cam1Δ* mutant strains (Fig. 3A). In S. pombe, Ncs1 physically interacts with the Mid1 ortholog, Yam8, which is a stretch-activated Ca^2+^ channel. S. pombe *YAM8* gene disruption in the *ncs1Δ* background restored the Ca^2+^-sensitive phenotype (35). In this study, the authors proposed that Ncs1 negatively regulates the Yam8 calcium channel. We therefore investigated whether the high-affinity Mid1-Cch1 calcium channel complex (48, 49) is responsible for the increased intracellular Ca^2+^ observed in the *ncs1Δ* mutant. Specifically, we used real-time reverse transcription-quantitative PCR (RT-qPCR) to compare the expressions of *MID1* and *CCH1* in WT and *ncs1Δ* grown in yeast extract-peptone-dextrose (YPD) with or without 100 mM CaCl_2_ for 24 h. The transcript levels of both genes increased by approximately 3-fold in the *ncs1Δ* mutant strain, but only following growth in the presence of 100 mM CaCl_2_ (Fig. 3B). No differences in *CCH1* and *MID1* expression was observed in the WT and the *ncs1Δ* strain grown in YPD without CaCl_2_ (Fig. 3B), suggesting that the Mid1-Cch1 complex, which imports Ca^2+^ into the cytosol (29, 48), is not the source of extra Ca^2+^ in the *ncs1Δ* mutant. In further support of this, we generated a *mid1Δ ncs1Δ* double mutant in C. neoformans and found that increased intracellular Ca^2+^ accumulation and Ca^2+^ sensitivity persisted in this mutant (Fig. S3). Despite these findings, we cannot rule out the involvement of other low-affinity calcium channels in contributing to the increased intracellular Ca^2+^ observed in the *ncs1Δ* mutant.

10.1128/mSphere.00761-20.5 FIG S3 Disruption of *MID1* does not rescue the *ncs1Δ* calcium-sensitive phenotype. (A) Spot dilution assay was performed for the WT, *ncs1Δ* and *mid1Δ ncs1Δ* strains in the presence of increasing CaCl_2_ concentrations. Pictures were taken after 48 h of incubation at 30°C. (B) The basal level of free intracellular Ca2^+^ in the WT, *ncs1Δ*, *ncs1Δ*::*NCS1*, *mid1Δ ncs1Δ*, *cna1Δ*, and *cam1Δ* strains was quantified by flow cytometry following staining with the calcium-specific dye Fluo-4-AM. The left side represents the histogram of Fluo-4-AM emitted fluorescence of the strains cultivated in YPD medium at 30°C. The right side represents the percentage of gated fluorescent cells ± standard deviation (three biological replicates). Mean values were compared using one-way ANOVA and Dunnett’s *post hoc* for statistical analysis. ****, *P* < 0.0001; *, *P* < 0.05. Download FIG S3, TIF file, 2.4 MB. Copyright © 2020 Squizani et al. 2020 Squizani et al. This content is distributed under the terms of the Creative Commons Attribution 4.0 International license.

We also C-terminally tagged Ncs1 with green fluorescent protein (GFP) (*NCS1*::*GFP*) to assess Ncs1 subcellular localization. Faint, predominantly cytosolic, Ncs1 fluorescence was observed when the strain was cultured in the absence of Ca^2+^. However, the fluorescence was higher than that observed for the nonfluorescent WT control strain (Fig. 3C). Ncs1 fluorescence became more intense following culture in the presence of Ca^2+^ (100 mM CaCl_2_), with Ncs1 adopting a more punctate staining pattern: 24.5% ± 1.1% and 36.3% ± 3.0% of the cell population displayed puncta in the absence (MM) and presence (MM + CaCl_2_) of Ca^2+^, respectively (*P* = 0.013, Welch’s test with ≥200 cells per sample) (Fig. 3C). Increased Ncs1 fluorescence in the presence of Ca^2+^ correlated with higher expression of *NCS1* by the *NCS1*::*GFP* strain under the same condition (Fig. 3D). Taken together, these results suggest that Ncs1 responds to increase in intracellular Ca^2+^ levels and participates in the regulation of calcium homeostasis in C. neoformans.

### *NCS1* is a calcineurin-Crz1 responsive gene.

**FIG 4:**
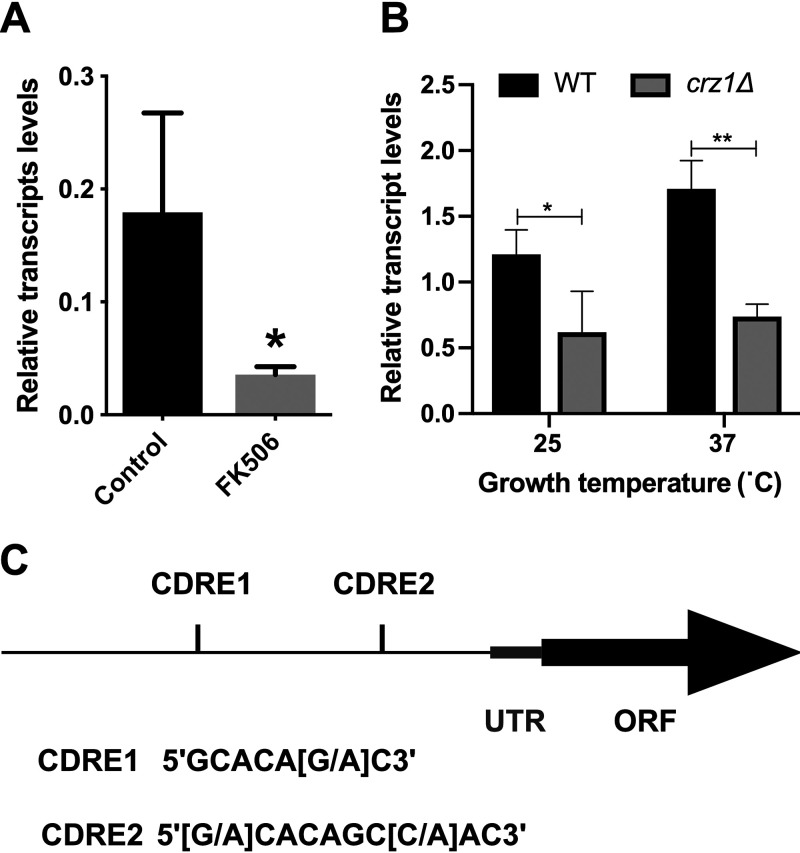
*NCS1* gene expression is regulated by Crz1. (A) The transcript levels of *NCS1* were determined under conditions of calcineurin inhibition. Yeast cells were incubated in YPD medium at 37°C in the absence or presence of FK506 (1 μg/ml) for 1 h. *NCS1* expression was normalized to *ACT1* transcript levels. Bars represent the means ± standard deviations (three biological replicates). Statistics were conducted using Student’s *t* test (*, *P* < 0.05; ***, *P < *0.001). (B) *NCS1* gene expression in WT and *crz1Δ* null mutant cells was assessed by RT-qPCR. Yeast cells were incubated in YPD at 25°C or 37°C for 16 h. *NCS1* expression was normalized to *ACT1* transcript levels. Each bar represents the mean ± the standard deviation (three biological triplicates). Statistical analysis was performed using Student’s *t* test (*, *P < *0.05; **, *P < *0.01). (C) The *NCS1* regulatory sequence contains two Crz1 binding motifs (CDRE1 and CDRE2). CDRE, calcineurin-dependent response element; UTR, untranslated region; ORF, open reading frame.

Given that *NCS1* is a Ca^2+^-responsive gene in C. neoformans (Fig. 3D), we investigated whether *NCS1* expression is regulated by the calcineurin signaling pathway via the transcription factor Crz1. *NCS1* expression was analyzed in the presence and absence of the calcineurin inhibitor FK506 (Fig. 4A) and in the WT and *crz1*Δ mutant (Fig. 4B). The results demonstrated that FK506 treatment reduced *NCS1* transcription in the WT (Fig. 4A) and that *NCS1* expression was downregulated in the *crz1*Δ mutant at 25°C and 37°C (Fig. 4B). In further support of *NCS1* being a Crz1 target, we identified two Crz1-binding consensus motifs (50) in the putative *NCS1* regulatory region encompassing the 1,000-nucleotide sequence upstream of the transcription start site (Fig. 4C). These findings provide evidence that Ncs1 and calcineurin work together to regulate Ca^2+^ homeostasis.

### Ncs1 activity is essential for C. neoformans virulence.

**FIG 5:**
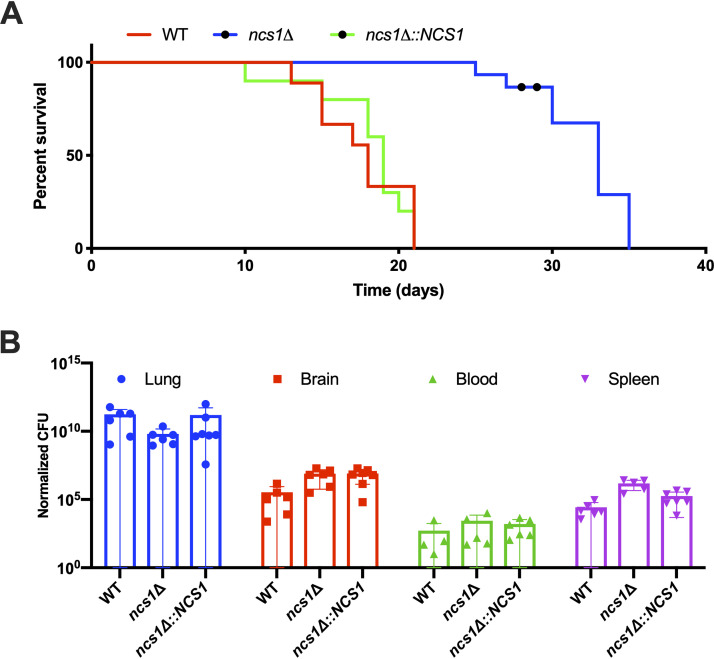
Ncs1 is required for full virulence in a mouse inhalation model of cryptococcosis. C57BL/6J mice (10 mice per group) were infected with 500,000 cells of the WT, *ncs1Δ*, or *ncs1Δ*::*NCS1* strain. Mice were monitored daily and euthanized by CO_2_ asphyxiation when they had lost 20% of their preinfection weight. (A) Median mouse survival differences were estimated using a Kaplan-Meier log-rank Mantel-Cox test. The increase in median survival of *ncs1Δ-*infected mice relative to the other two infection groups was statistically significant (*P < *0.0001). (B) Lungs, brain, and spleen were removed posteuthanasia, weighed, homogenized, serially diluted, and plated onto Sabouraud dextrose agar plates to determine fungal burden by quantitative culture (CFU) following 3 days of growth at 30°C. CFU were adjusted to reflect CFU/gram of tissue and CFU/milliliter of blood (normalized CFU). Statistical significance was determined using one-way ANOVA. However, no differences in organ burden were found.

As proven in other studies, the disruption of Ca^2+^ homeostasis components is important for cryptococcal pathogenicity (29–33, 51). To determine whether disruption of Ncs1-mediated calcium homeostasis also contributes to pathogenicity, we compared the virulence of the *ncs1Δ* mutant strain to that observed for the WT and *ncs1Δ*::*NCS1* strains in a mouse inhalation model of cryptococcosis. In a Kaplan-Meier survival study, the *ncs1Δ* null mutant strain was found to be hypovirulent (median lethal time [LT_50_], 32.7 days) compared to the WT (LT_50_, 18.9; *P* < 0.0001) and the *ncs1Δ*::*NCS1* strain (LT_50_, 17.4 days; *P* < 0.0001) (Fig. 5A). Although the disruption of *NCS1* prolonged mouse survival, no difference in the fungal burdens in lung and brain were observed at time of death, when infected mice had lost 20% of their preinfection weight (Fig. 5B). Thus, the *ncs1Δ* null mutant strain is capable of infecting the lung and brain tissue but potentially grows at a lower rate than the WT and the *ncs1Δ*::*NCS1* strains.

### Ncs1 is necessary for growth under host-mimicking conditions.

**FIG 6:**
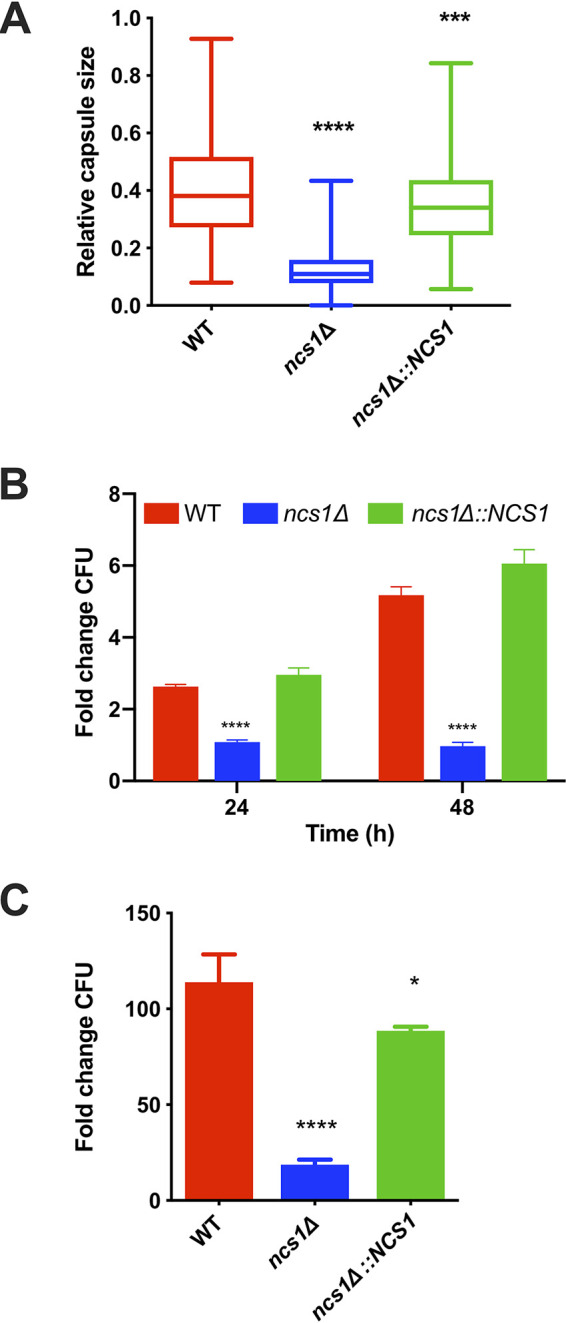
Ncs1 is necessary for growth under host-mimicking conditions. (A) Capsule sizes of WT, *ncs1Δ*, and *ncs1Δ*::*NCS1* cells were determined following incubation in capsule-inducing medium (DMEM) for 72 h (37°C and 5% CO_2_). Capsules were visualized by India ink staining and light microscopy, and measurements were performed using ImageJ software for at least 50 cells of each strain. Relative capsule size was defined as the distance between the cell wall and the capsule outer border by cell diameter. Statistical analysis was performed using one-way ANOVA, with Tukey *post hoc* test. **** *P < *0.0001, and ***, *P < *0.001, compared to the WT. (B) Growth of the WT, *ncs1Δ*, and *ncs1Δ*::*NCS1* cells in DMEM (37°C and 5% CO_2_) for 24 or 48 h was assessed by quantitative culture (CFU). The results represent the mean ± standard deviation (three biological replicates) of each strain normalized to the CFU of the inoculum, described as fold change. Statistical analysis was performed using one-way ANOVA with Dunnett’s *post hoc* test. Significant differences compared to WT are marked (****, *P < *0.0001). (C) Growth of the WT, *ncs1Δ*, and *ncs1Δ*::*NCS1* cells for 24 h at 37°C 5% CO_2_ in heat-inactivated mouse serum was by quantitated (CFU). The results are expressed as a fold change relative to the initial inoculum (10^4^ cells/ml) and represent the means ± standard deviations (three biological replicates). Statistical analysis was performed using one-way ANOVA and Dunnett’s *post hoc* test (*, *P < *0.05, and ****, *P < *0.0001, relative to the WT).

We also analyzed the capability of *ncs1Δ* mutant to synthesize the polysaccharide capsule, since this is the main cryptococcal virulence factor (1, 12). We observed that when the *ncs1Δ* strain was grown under capsule-inducing conditions (Dulbecco modified Eagle medium [DMEM] at 37°C and 5% CO_2_), mutant cells produced smaller capsules than the WT and *ncs1Δ*::*NCS1* strain (Fig. 6A). However, capsule size was not affected following growth in mouse serum (data not shown). Next, we compared growth of the *ncs1Δ* mutant to that of the WT and *ncs1Δ*::*NCS1* strains under the capsule induction condition utilized and found that the null mutant growth was drastically compromised (Fig. 6B). Similarly, growth of the *ncs1Δ* strain was severely impaired in mouse serum over a 24-h period at 37°C with 5% CO_2_ (Fig. 6C). Collectively, these results suggest that hypovirulence of the *ncs1Δ* mutant is most likely associated with the observed growth defects, with reduced capsule size making only a minor contribution to this virulence phenotype.

### Ncs1 is important for the release of daughter cells.

**FIG 7:**
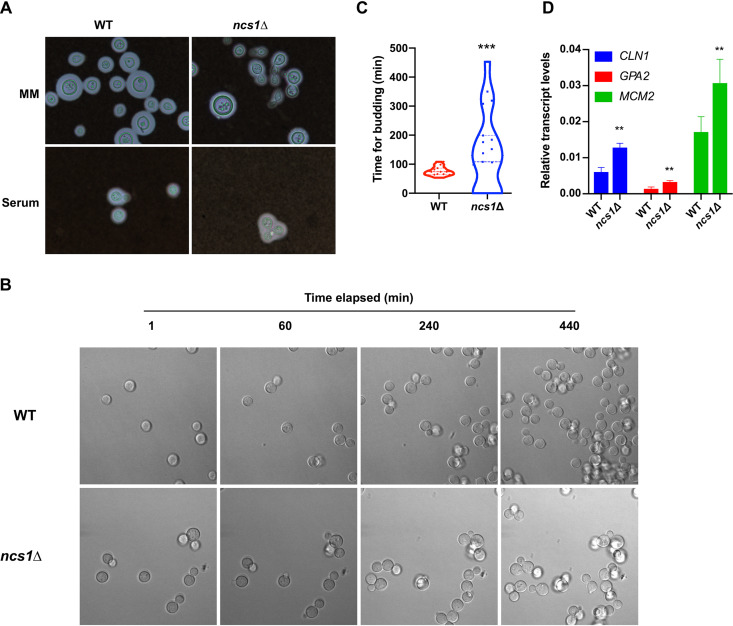
The *ncs1Δ* null mutant strain displays aberrant cell division and morphology (A), delayed bud emergence (B and C), and altered cell cycle regulation (D). (A) WT and *ncs1Δ* cells were grown in minimal medium for 72 h at 37°C and 5% CO_2_ (upper panel) or in heat-inactivated mouse serum for 24 h at 37°C and 5% CO_2_ (lower panel), stained with India ink, and visualized by light microscopy. (B and C) Fungal cells were incubated in YPD medium for 16 h inside a chamber coupled to a confocal microscope (37°C and 5% CO_2_), and bud emergence time was recorded using time-lapse microscopy. Time measurements were initiated after the first round of bud emergence to avoid errors associated with the lack of synchronization. Images were acquired every 30 s. The graph in panel C represents the mean time for buds to emerge (minutes) ± standard deviation of at least 15 cells per strain. Statistical analysis was performed using the nonparametric Mann-Whitney *test* (***, *P* < 0.0001). (D) Transcript levels of genes encoding cell cycle regulators were assessed in WT and *ncs1Δ* cells by RT-qPCR. Cells were grown in YPD at 37°C for 4 h. Results represent the mean transcript levels ± standard deviations (three biological triplicates) with each gene normalized to *ACT1* transcript levels. Statistical analysis was performed using Student’s *t* test (**, *P < *0.01).

Microscopic analysis to evaluate the size of the polysaccharide capsule and the growth rate in mouse serum revealed that some *ncs1Δ* cells displayed aberrant morphology and cell division (Fig. 7A), suggesting that Ncs1 could play a role in cell cycle progression. We therefore investigated the growth defect further by determining the time it took for buds to emerge using time-lapse microscopy (Fig. 7B and Movies S1 and S2). Given that the mutant was severely attenuated in growth when cultured in DMEM or exposed to mouse serum, we chose YPD medium for this analysis, as it is a richer medium in which mutant growth is not as compromised. To avoid bias due to lack of synchronization, we only measured the time of bud emergence in cells after the bud of the first daughter cell had separated from the mother or, in the case of the mutant cells, where progeny did not detach from mother cell, after the second bud emergence. The results demonstrate that it took ∼70 min for buds to emerge in the WT cells and more than 140 min for buds to emerge in isolated and clumped *ncs1*Δ mutant cells (Fig. 7C). Furthermore, buds were slow to be released in some *ncs1*Δ mutant cells, resulting in more extensive cell clumping.

10.1128/mSphere.00761-20.1 MOVIE S1 Time-lapse video microscopy demonstrating WT bud emergence profile. Download Movie S1, MOV file, 9.9 MB. Copyright © 2020 Squizani et al. 2020 Squizani et al. This content is distributed under the terms of the Creative Commons Attribution 4.0 International license.

10.1128/mSphere.00761-20.2 MOVIE S2 Time-lapse video microscopy demonstrating *ncs1Δ* bud emergence profile. Download Movie S2, MOV file, 1.4 MB. Copyright © 2020 Squizani et al. 2020 Squizani et al. This content is distributed under the terms of the Creative Commons Attribution 4.0 International license.

As cell division is linked to the cell cycle, we evaluated whether cells lacking *NCS1* displayed defects in cell cycle regulation by measuring the levels of two transcripts associated with different stages of the cell cycle: the G_1_ cyclin encoded by *CNL1* (52) and the S phase DNA replication licensing factor encoded by *MCM2* (53, 54). We also measured the transcript levels of the G protein-coupled receptor encoded by *GPA2*, which displays oscillatory expression during the cell cycle (53, 54). All three genes were upregulated in the *ncs1Δ* strain compared to the WT after 4 h of growth in YPD (Fig. 7D), reinforcing that cell cycle progression is altered in the *ncs1Δ* mutant strain.

## DISCUSSION

Our results indicate that *NCS1* expression in C. neoformans is regulated by Ca^2+^ and the calcineurin/Crz1 pathway and corroborate findings on the Ncs1 ortholog in fission yeast (35). In contrast to our conclusions and those made in studies using S. pombe, the S. cerevisiae Ncs1 ortholog, Frq1, was found to be essential for viability and the level of *FRQ1* expression was not influenced by the calcineurin/Crz1 pathway, as revealed by microarray analysis (55). This suggests that distinct calcium sensing mechanisms exist in fungal species despite widespread functional conservation of Ncs1 and other regulators of Ca^2+^ homeostasis.

An interesting feature of the *ncs1Δ* mutant is its attenuated virulence in a murine model of cryptococcosis. In contrast, attenuated virulence was not observed for the null Ncs1 ortholog mutant (*NCSA*) in A. fumigatus (41), reaffirming that processes regulated by Ncs1 orthologs in pathogenic fungi differ or that other genes can compensate in the absence of *NCSA*. Moreover, the cryptococcal *ncs1Δ* strain took longer to achieve the growth densities associated with debilitating infection in the tissues of WT-infected mice. This slower growth phenotype *in vivo* correlated with the reduced rate of proliferation of the *ncs1Δ* strain in mouse serum and impaired bud emergence and release. These results confirm that Ncs1 is important for fungal adaptation to the host environment and for the establishment of disease and reaffirm the importance of Ca^2+^ homeostasis and Ca^2+^ signaling in cryptococcal virulence. Our findings also extend the set of calcium-related genes involved in virulence to include Ncs1.

Given that expression of ∼40 virulence-associated genes is linked to the cell cycle in C. neoformans, the control of this process is fundamental to disease progression (53). In S. cerevisiae, Ca^2+^ homeostasis is linked to cell cycle regulation, as a decrease in intracellular Ca^2+^ leads to transient arrest in the G_1_ phase, followed by interruption in the G_2_/M phase (27, 56–58). Moreover, bud emergence and the cell cycle depend on calcineurin activity, which regulates the availability of proteins involved in cell cycle regulation. These proteins include Swe1, a negative regulator of Cdc28/Clb complex, Cln2, a protein kinase required for cell cycle progression, and a G_2_ cyclin (27). In this context, we speculate a potential role for calcineurin signaling in cell cycle regulation in C. neoformans. Our new data demonstrate that Ncs1 interferes with the transcription profile of genes associated with cell cycle progression (*CLN1*, *GPA2*, and *MCM2*). Notably, overexpression of the S. cerevisiae G_1_/S cyclin, Cln1, led to a filamentation phenotype (59). In our study, we showed that the cryptococcal *ncs1*Δ mutant was impaired in bud emergence and release, as seen in time-lapse microscopy. Furthermore, the C. neoformans *cln1Δ* mutant exhibited aberrant bud emergence and cell division and a consistent delay in budding (60), which are phenotypes also observed in cryptococcal *ncs1Δ* mutant cells. Additional experiments to confirm the impact of Ca^2+^ homeostasis on cryptococcal cell cycle regulation are necessary to support this hypothesis.

We hypothesize that Ca^2+^ excess, or even other types of stress, leads to activation of the calcineurin pathway, which ultimately drives the expression of Ncs1 in a Crz1-dependent fashion. Therefore, Ca^2+^-activated Ncs1 would participate in a diverse array of cellular processes to cope with Ca^2+^ excess, including the regulation of cell division via its potential association with Pik1, a protein implicated in cell septation in fission yeast (61). Two lines of evidences support this hypothesis: (i) yeast Pik1 forms puncta consistent with its localization in the Golgi apparatus (62) and we observed that cryptococcal Ncs1 also forms puncta, particularly when Ca^2+^ is present, and (ii) yeast Frq1 physically interacts with Pik1 (62). Structural studies performed on Ncs1 revealed that the myristoyl group flips out following Ca^2+^ binding, allowing Ncs1 to anchor reversibly to membranes (20, 39, 40). Thus, it is possible that Ca^2+^ binding to Ncs1 exposes the hydrophobic N-myristoylation domain, promoting Ncs1 association with Pik1 in Golgi membranes and, hence, proper cell septation and division. Further experiments are required to confirm that the puncta assumed by Ncs1 upon addition of calcium colocalize with the Golgi.

In summary, we have characterized the Ncs1 homolog in C. neoformans, demonstrating its importance in Ca^2+^ homeostasis and virulence. We showed that in contrast to S. cerevisiae, *NCS1* is a calcineurin-responsive gene in C. neoformans, with calcineurin and Ncs1 working together to regulate calcium homeostasis and, hence, promote fungal growth and virulence. To our knowledge, this is the first report of a role for Ncs1 in fungal virulence using a mammalian infection model and of a potential correlation between Ca^2+^ signaling and cell cycle progression in C. neoformans.

## MATERIALS AND METHODS

### Fungal strains and media.

C. neoformans serotype A strain Kn99 was chosen to conduct the study as the wild type (WT). The *NCS1* gene (CNAG_03370) deletion mutant (*ncs1*Δ), *cna1Δ* mutant, and *cam1Δ* mutant were obtained from H. Madhani’s library (47). The *ncs1*Δ reconstituted strain (*ncs1Δ*::*NCS1*), the *mid1*Δ *ncs1*Δ double mutant, and the *NCS1*::*GFP* strain were all constructed using overlapping PCR as previously described (63), and site-directed homologous recombination was performed. Transformation was carried out using biolistic transformation, as previously described (64). The primer list is presented at Table S1, and the confirmations of the cassette’s insertions are demonstrated in Fig. S1. Fungal cells were maintained on solid YPD medium (1% yeast extract, 2% peptone, 2% dextrose, and 1.5% agar). YPD plates containing hygromycin (200 μg/ml) or G418 (100 μg/ml) were used to select C. neoformans transformants.

10.1128/mSphere.00761-20.6 TABLE S1 List of primers used in this study. Download Table S1, DOCX file, 0.02 MB. Copyright © 2020 Squizani et al. 2020 Squizani et al. This content is distributed under the terms of the Creative Commons Attribution 4.0 International license.

### *In silico* analysis.

To evaluate Ncs1 protein conserved domains, we used the protein sequences and annotations retrieved from the FungiDB database (http://fungidb.org) (65), applying FungiDB tools and the InterproScan database (66). The same was performed for NMT-themyrpredictor database to identify the N-terminal myristoylation consensus sequence. Conservation was assessed using BLASTp against target proteins. Finally, the presence of the Crz1‐binding motif on the promoter region of the *NCS1* gene was made by manual search. We recovered the putative regulatory regions of cryptococcal genes from FungiDB (http://fungidb.org), selecting 1,000 bp upstream of the transcription start site of *NCS1* gene. The sequences utilized for Crz1‐binding motif search are already described (50).

### Virulence assay.

Virulence assays were performed as previously described (67). Briefly, female C57BL/6 mice (10 per infection group) were anesthetized by inhalation of 3% isoflurane in oxygen and infected with 5 × 10^5^ fungal cells (WT, *ncs1Δ*, or *ncs1Δ*::*NCS1* strain) via the nasal passages. Mice were monitored daily and euthanized by CO_2_ asphyxiation when they had lost 20% of their preinfection weight or prior in the case of debilitating symptoms of infection. Median survival differences were estimated using a Kaplan-Meier log-rank Mantel-Cox test. Posteuthanasia, lungs and brain were removed, weighed, and homogenized in 2 ml of sterile phosphate-buffered saline (PBS) using a BeadBug (Benchmark Scientific). Organ homogenates were serially diluted and plated onto Sabouraud dextrose agar plates. Plates were incubated at 30°C for 2 days. Colony counts were performed and adjusted to reflect the total number of CFU per gram of tissue or milliliter of blood. For fungal burden analysis, two-way analysis of variance (ANOVA) with Tukey *post hoc* was utilized to determine the statistical significance.

### C. neoformans replication in mouse serum.

A total of 10 BALB/c mice (10 weeks old) were obtained from Biotechnology Center, UFRGS, Brazil. Mice were anesthetized using isoflurane (in a chamber), and blood was collected from the retro-orbital space, using a glass capillary. Next, mice were euthanized using an overdose of thiopental (140 mg/kg of body weight). Serum was obtained from total blood after centrifugation (3,000 × *g* for 15 min at room temperature). A total of 1,000 cells in 100-μl suspensions of the WT, *ncs1Δ*, and *ncs1Δ*::*NCS1* strains were inoculated in heat-inactivated mouse serum in a 96-well plate and incubated at 37°C and 5% CO_2_ for 24 h. Yeast cells were collected and plated on YPD plates for CFU determination. Separate wells were used for cell morphology analysis, in which yeast cells were first fixed with 4% paraformaldehyde for 30 min at 37°C and then analyzed using light microscopy.

### Yeast growth in DMEM.

A total of 1 × 10^6^ cells in 1,000-μl suspensions of the WT, *ncs1Δ*, and *ncs1Δ*::*NCS1* strains were inoculated in DMEM in a 24-well plate and incubated at 37°C and 5% CO_2_ for 24 and 48 h. Next, yeast cells were gathered and plated on YPD plates for CFU determination. Separate wells were used for cell morphology analysis, in which fungal cells were fixed with 4% paraformaldehyde for 30 min at 37°C and then analyzed using India ink counterstaining in light microscopy.

### Intracellular calcium measurements.

Free intracellular Ca^2+^ in C. neoformans was quantified by flow cytometry (Millipore GuavaSoft) following cellular staining with the calcium sensor dye Fluo-4-AM (Thermo Fisher Scientific) at a final concentration of 2 μM. Briefly, yeast cells were cultured overnight on YPD at 30°C with shaking. Next, cells were centrifuged (6,000 rpm for 3 min) and washed twice with PBS. After adjusting the cell density (optical density at 600 nm [OD_600_] = 1.0), the Fluo-4-AM dye was added to each tube and incubated at 37°C for 1 h. The flow was adjusted to pass <500 cells/μl, and a total of 5,000 events were evaluated.

### Phenotypic characterization.

For phenotypic characterization, WT, *ncs1Δ* mutant, and *ncs1Δ*::*NCS1* complemented strains were grown overnight on YPD at 30°C with shaking. Further, cells were centrifuged, washed twice with deionized water, and adjusted to 10^8^/ml. The cell suspensions were then subjected to serial dilution (10-fold), and 3 μl of each dilution was spotted onto YPD agar supplemented with different stressors, including CaCl_2_ (200 mM and 300 mM). Cell wall perturbation was assessed using Congo red (0.1%) and calcofluor white (0.5 mg/ml), as previously described (24). The sensitivity to osmotic stress was evaluated utilizing NaCl at 1 M. Moreover, menadione (30 μM) was used as an oxidative stressor, and a low-phosphate environment was used as a starvation condition (68). All the plates were incubated for 48 h at 30°C and photographed, with the exception of plates incubated at high temperatures (37°C or 39°C).

### Fluorescence and light microscopy.

Fluorescence microscopy assays were accomplished using a DeltaVision fluorescence microscope. WT and *NCS1*::*GFP* cells were incubated in minimal medium (2 g/liter of l-asparagine, 1 g/liter of MgSO_4_· 7H_2_O, 6 g/liter of KH_2_PO_4_, 2 g/liter of thiamine) without or supplemented with 100 mM CaCl_2_ for 16 h at 30°C with shaking. Thereafter, cells were washed once with PBS and analyzed. For light microscopy, WT, *ncs1Δ* mutant, and *ncs1Δ*::*NCS1* cells were grown in DMEM or minimal medium at 37°C and 5% CO_2_ for 72 h. Next, the cells were fixed with 4% paraformaldehyde for 30 min at 37°C, washed with PBS, and then analyzed under light microscopy, using counterstaining with India ink. To define the relative capsule sizes, measures of the distance between the cell wall and the capsule outer border were determined and divided by each cell diameter through ImageJ software (https://imagej.nih.gov/ij/). At least 50 cells of each strain were measured.

### Time-lapse microscopy.

Cellular division was followed using confocal microscopy. The experimental design was as already described (52), with few modifications. Briefly, WT or *ncs1Δ* mutant cells were cultured overnight on liquid YPD medium at 30°C with shaking. Further, cells were washed twice with PBS and adjusted to 10^6^/ml with YPD medium at pH 7.45. One hundred microliters of cell suspension was inoculated on a 35/10-mm glass-bottom cell culture dish (Greiner Bio-one). The culture dish was previously treated with 100 μl of poly-l-lysine (0.1 mg/ml) for 1 h, washed 3 times with PBS, and then incubated with 10 μg/ml of monoclonal antibody (MAb) 18B7 for 1 h. The culture dishes were incubated in a temperature-controlled microscope chamber adjusted to 37°C and 5% CO_2_. Image acquisition was done in a 30-s interval, using a differential inference contrast (DIC) objective in an FV1000 confocal microscope at the Microscopy and Microanalysis Center (CMM) of the Universidade Federal do Rio Grande do Sul (UFRGS). Statistical analysis was done by timing how long each mother cell took to originate a bud. Measurements were performed at the beginning of the second budding in order to avoid errors associated with the lack of tools to synchronize cells.

### RT-qPCR analysis.

For gene expression analysis, strains were subjected to different conditions, as described in the figure legends. RT-qPCR technique was performed for all experiments as follows. Cryptococcal cells were washed once with PBS, then frozen in liquid nitrogen, and lyophilized. Cell lysis was performed by vortexing the tubes with the dry pelleted cells using acid-washed glass beads (Sigma-Aldrich Co., St. Louis, MO). Three independent sets of RNA samples for each strain were prepared using TRIzol reagent (Invitrogen, Carlsbad, CA) according to the manufacturer’s protocol. Next, RNA samples were treated with DNase (Promega, Madison, WI), and a total of 300 ng of treated-RNA was used for reverse transcription with ImProm-II reverse transcriptase (Promega). RT-qPCR was performed on a real-time PCR StepOne real-time PCR system (Applied Biosystems, Foster City, CA). PCR thermal cycling conditions had an initial step at 94°C for 5 min, followed by 40 cycles at 94°C for 30 s, 60°C for 30 s, and 72°C for 60 s. Platinum SYBR green qPCR Supermix (Invitrogen, Carlsbad, CA) was used as the reaction mix, with 1 μl of the cDNA (16 ng) template, in a final volume of 20 μl. Each cDNA sample was done in technical triplicates. Melting-curve analysis was performed at the end of the reaction to confirm a single PCR product. The data were normalized to the actin cDNA levels. Relative expression was determined by the threshold cycle (2^−Δ^*^CT^*) method (69).

### Ethics statement.

The animals were obtained from the Animal Resource Centre, Floreat Park, Western Australia, Australia. The *in vivo* procedures were performed under protocol number 4254, approved by Western Sydney Local Health District Animal Ethics Committee, accomplished according to the current guidelines of The National Health and Medical Research Council of Australia. The Animal Use Ethics Committee (CEUA/UFRGS) approved the animal experimentation under reference no. 22488.
