# Supplementary figures and images for: Calcium Binding Protein Ncs1 Is Calcineurin Regulated in Cryptococcus neoformans and Essential for Cell Division and Virulence
Source: mSphere. 2020 Sep 9;5(5):e00761-20. doi: 10.1128/mSphere.00761-20 (PMC7485688; doi:10.1128/mSphere.00761-20)

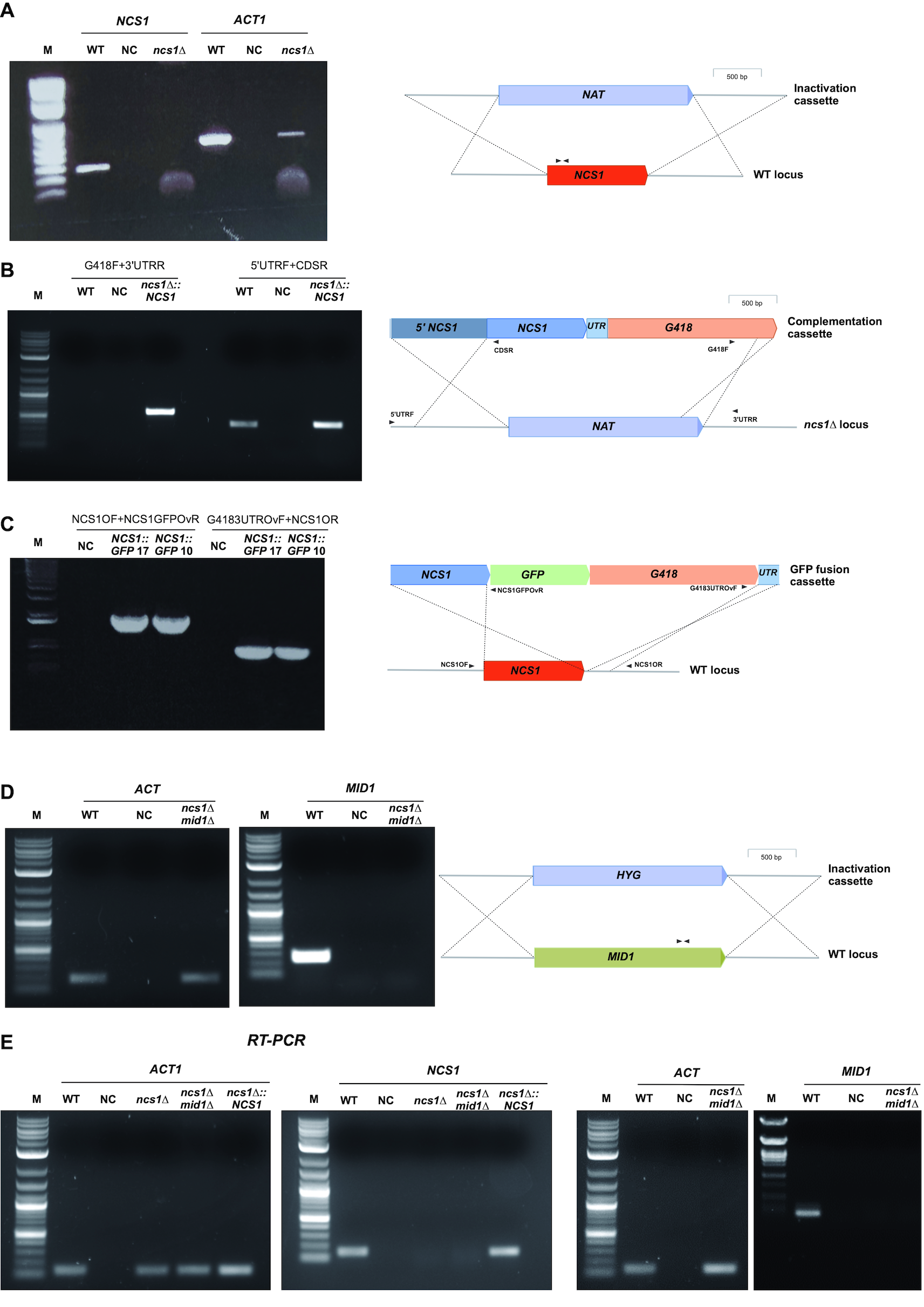

Supplement: FIG S1 [file mSphere.00761-20-sf001.tif]

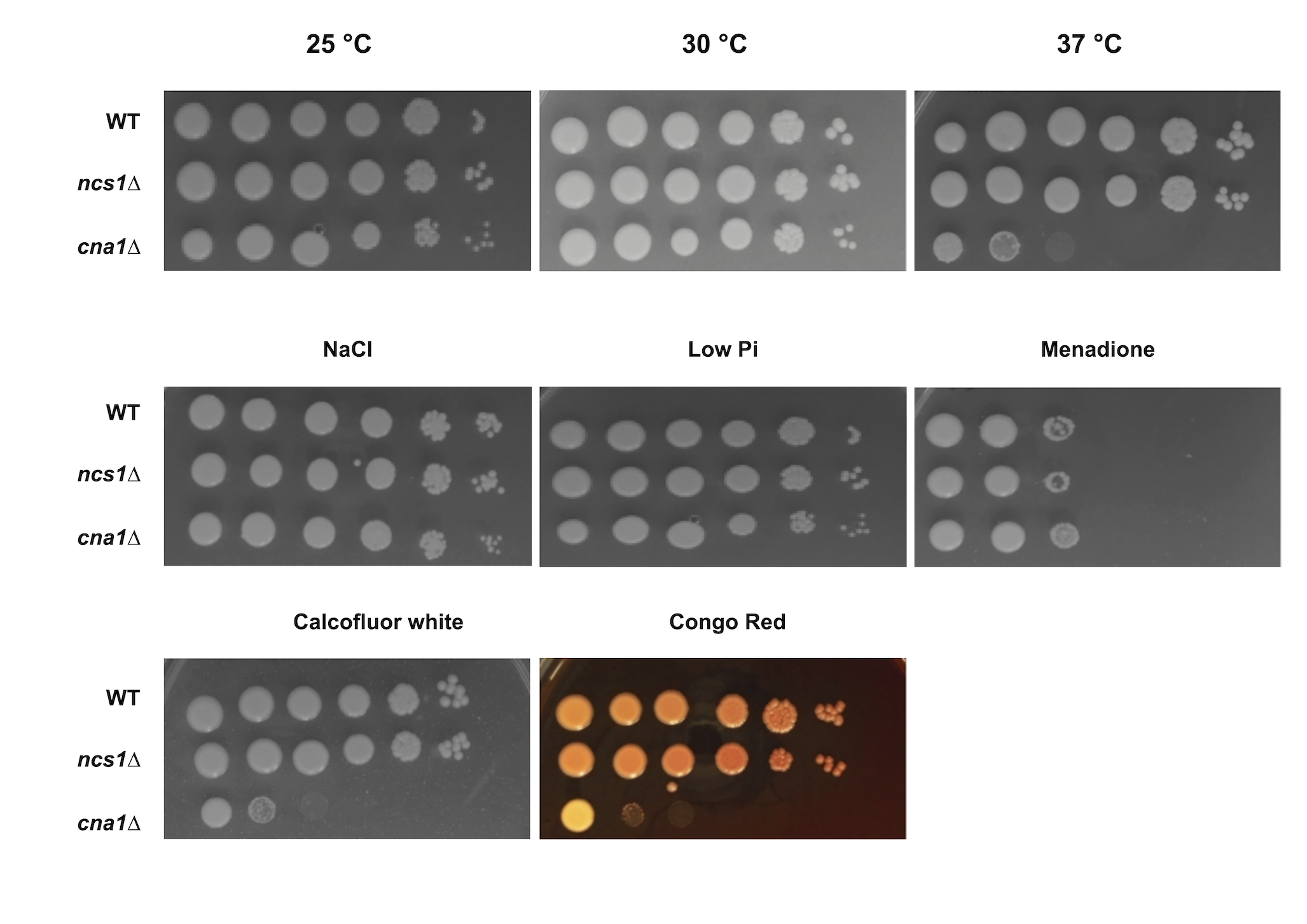

Supplement: FIG S2 [file mSphere.00761-20-sf002.tif]

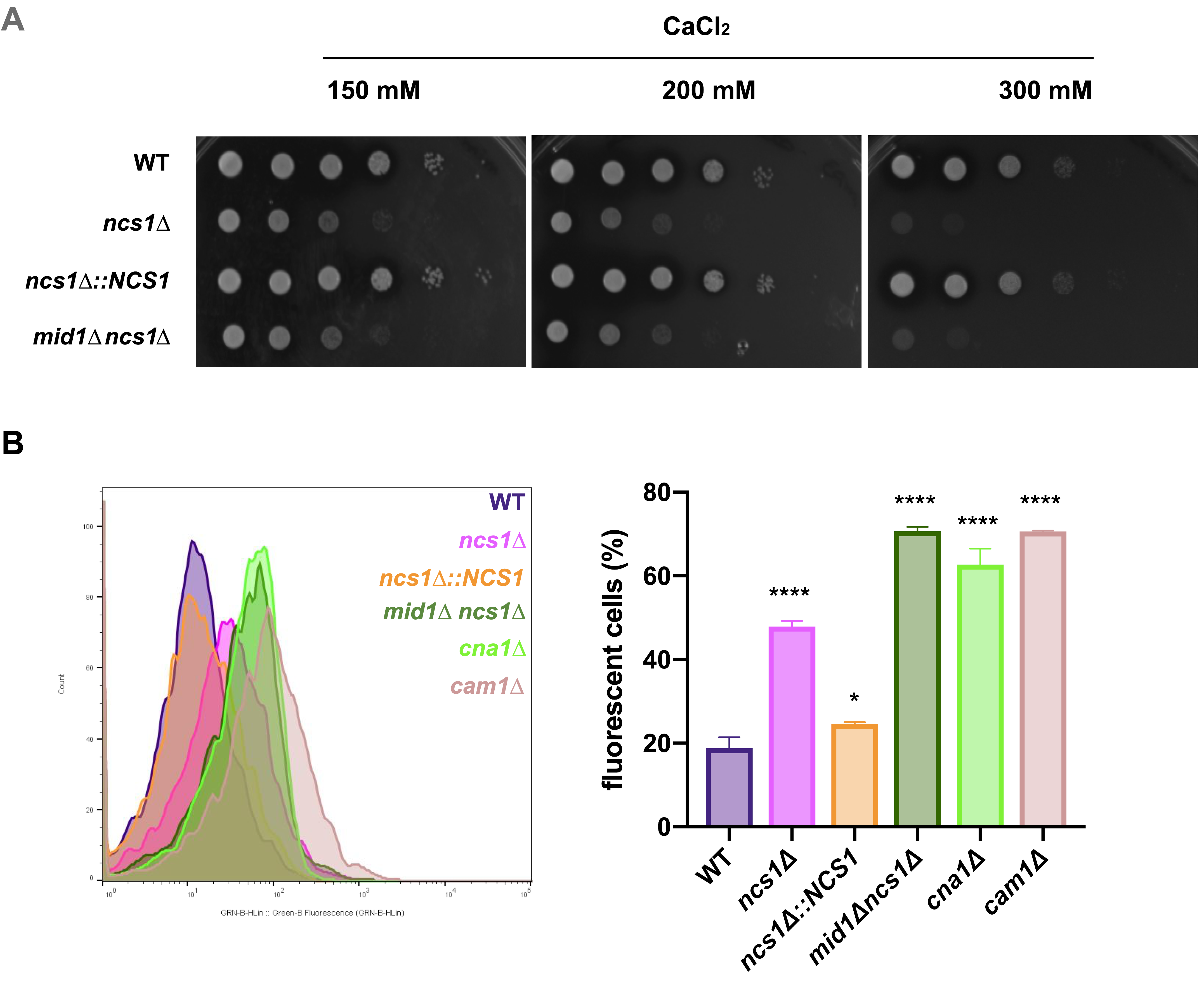

Supplement: FIG S3 [file mSphere.00761-20-sf003.tif]
