# Supplementary material for: Calcium Binding Protein Ncs1 Is Calcineurin Regulated in Cryptococcus neoformans and Essential for Cell Division and Virulence
Source: mSphere. 2020 Sep 9;5(5):e00761-20. doi: 10.1128/mSphere.00761-20 (PMC7485688; doi:10.1128/mSphere.00761-20)
Supplement: TABLE S1 [file mSphere.00761-20-st001.docx]

| **Primer** | **Primer Sequence (5’- 3’)** |
| --- | --- |
| ***MID1 disruption*** | |
| MID1_5UTRF | GCCTCTTTTTCCTTTTCTGT |
| MID1_5UTRR | CACTGGCCGTCGTTTTACTTGCCTGTTCGCTATCGTCT |
| MID1_3UTRF | CATGGTCATAGCTGTTTCCTGAATAGAGGTGTGTGATTGA |
| MID1_3UTRR | ATACGCTGGGAAAGGTGTCT |
| **Marker amplification for Split-marker** | |
| SM1 | CGAAGAATCTCGTGCTTTC |
| SM2 | ATTGACCGATTCCTTGCG |
| ***NCS1* amplification for complemented strain *ncs1∆::NCS1*** | |
| NCS1_RECF | CCCAGACTCACCACATATCAACATC |
| NCS1_RECR | CTCCAGCTCACATCCTCGCAGATAATTTGCATGCCATCGAC |
| **Amplification of selection marker G418 for complemented strain *ncs1∆::NCS1*** | |
| G418F | TCGATGGCATGCAAATTATCAGGATGTGAGCTGGAGAGCG |
| G418R | GGAGCCATGAAGATCCTGAG |
| **Generation of *NCS1::GFP*** | |
| NCS1OF | TACCATTCGATGACGGCCTG |
| INNER_5UTR_ NCS1F | ATCGGAGCAGGTGAACAGTG |
| NCS1GFPOVR | GAAGAGCTCCTCACCCTTGGACACCAACCCATCATACAGTG |
| NCS1OR | TGGGATTTACATTCCCCGCC |
| G4183UTROVF | TTTTAAATCTTGAATTTTAC |
| **RT-qPCR** |  |
| MID1F | GACGAGCAAGGATTTAGAAGC |
| MID1R | GCAGAGTAGCCCAGATTAGG |
| CCH1F | GCAAACTCCGTCCCAACTAC |
| CCH1R | GCACCTCGTTCAGCAGATTC |
| ACTF | CCTTCTACGTCTCTATCCAG |
| ACTR | TTTCAAGCTGAGAAGACTGG |
| NCS1F | GAAGATACTCCCGAAAAGC |
| NCS1R | TACACCAACCCATCATACAG |
| CLN1F | GGGGACTGAATGAATGGATG |
| CLN1R | TGAGGGCGTATGGAGAAAG |
| GPA2F | ATGGATCTGCCTTACAACAG |
| GPA2R | GTCTCTGGATGATTGATGG |
| MCM2F | TGCCCGCTTTTCTTCAGTC |
| MCM2R | TCGTCCTCGTCCATTCTCTC |
| **Diagnostic amplification of *NCS1∆::NCS1* cassette correct integration** | |
| G418F | GCCCATTAATCTATCTACAG |
| 3UTRR | GATCCAATCCCGACCGA |
| **Diagnostic amplification of *mid1∆* cassette correct integration** | |
| 5UTRF | GCAGCGACCAGGTTATTTA |
| CDSR | GGAGTGTACTCACAGTAAGT |
| CDSF | AACATCACCTATCCAGACA |
| 3UTRR | CGCTTGAGATTGAGAAT |
| 5UTRF | TCCCATTGTGGTCCTCG |
| **Diagnostic amplification of *NCS1::GFP* cassette correct integration** | |
| CDSR | TTTTGAACCGAGATTGAATG |
| NCS1OF | TACCATTCGATGACGGCCTG |
| NCS1GFPovR | GAAGAGCTCCTCACCCTTGGACACCAACCCATCATACAGTG |
| G4183UTRovF | TTTTAAATCTTGAATTTTAC |
| NCS1OR | TGGGATTTACATTCCCCGCC |
